# Chelsea Hospital for Women

**Published:** 1894-07-28

**Authors:** 


### The Institutional Workshop.

### CHELSEA HOSPITAL FOR WOMEN.

Abstract of a Report of the Committee of Inquiry in Regard to Statements made by Dr. Louis C. Parkes, the District Medical Officer of Health.

[The committee consisted of Lord Balfour of Burleigh, Lord Sandhurst, Sir Charles Dilke, Sir John Williams, and Messrs. H. G. Howse and Brass.]

We have now the honour to report the result of the inquiry which your lordship asked us to undertake in regard to the Chelsea Hospital for Women. Your lordship will recollect that on April 23rd you conveyed to us the request of the committee of the hospital that we should hold an inquiry concerning the statements made by Dr. Parkes to the vestry of the parish of chelsea concerning the hospital.

The statements referred to were embodied in a report to the vestry presented on February 6th by Dr. Parkes in his capacity as medical officer of health for the parish.

It appears to us that the statements made by Dr. Parkes in this report may be most conveniently dealt with under two main heads.

1st. That part of the report which deals with the general sanitary state of the hospital, and—

2nd. The reference to the mortality statistics of the patients in the hospital during the year 1893, with special regard to the deaths amongst the inmates upon whom surgical operations had been performed.

The committee first set forth the events which led up to the inquiry and give the correspondence in full.

The committee proceed: It will be observed that Dr. Parkes says the result of the examination into the condition of the drains of the hospital upon January 20th show that they leaked very seriously, and that other defects of an important nature were disclosed.

Mr. Wright, the treasurer of the hospital, demurs to the accuracy of these statements, and thinks that the comments made by Dr. Parkes upon the state of the sanitary arrangements of the hospital were unduly severe. We have no doubt that defects did exist which might have been, and probably were, the cause of serious evil, and we think Dr. Parkes was acting in accordance with his duty in calling attention to the matter, and the hospital authorities have since that time thoroughly overhauled the whole of the sanitary arrangements of the building, and it is represented to us that they have been put into as complete a sanitary condition as is possible at the present time.

In our opinion it was the plain duty of the medical officer of health to call the attention of the hospital authorities to the matter, and in the event of their having refused or shown any disinclination to attend to the matter with reasonable promptitude, it was undoubtedly his duty to make a formal report to the vestry on the subject We do not, however, understand upon what grounds Dr. Parkes, in presenting his report to the vestry, which contained the following paragraph: "It becomes my duty to recommend that notice should be served under the Public Health (London) Act upon the committee of the hospital, requiring them so to amend the sanitary arrangements as to place the hospital in a thorough sanitary condition within the space of two months, and to prohibit the use of the hospital to the public until such work has been performed to your satisfaction,' omitted to inform the vestry that at the time of presenting his report the hospital had actually been clear of patients for more than three weeks, and that some days previously to the presentation of his report he had actually received a letter from the secretary of the hospital, stating it was their intention to proceed with the work as soon as possible, and certainly before in-patients were readmitted to the building.

We now turn to the consideration of the second main head of the statements made by Dr. Parkes in his report to the vestry, that which has reference to the mortality statistics of the hospital and its causes.

We have thought it right to go very fully into the whole question of the operations which took place in the hospital during the year 1893, and we shall now proceed to set out the result at which we have arrived in regard to this portion of the inquiry.

For this purpose we have gone through the various statements contained in Dr. Parkes' letter of February 6th, comparing them with the tabulated statesments of Drs. Fenton and Eden, and with the reports of the cases in the books of the hospital.

The hospital contains sixty beds, into which 636 patients were admitted during the year 1893. (This number does not include the cases already in the hospital on January 1st.) Of these 636 thirty-seven died.

Three hundred and seventy-three patients were operated upon in the course of the year, of whom twenty-six died.

These operations fall under the following heads: (1) Removal of ovarian tumours (ovariotomy); (2) removal of uterine appendages (oophorectomy); (3) abdominal section for extra-uterine gestation; (4) abdominal hysterectomy for fibroid tumours; (5) abdominal section for removal of fibroid tumours (myomectomy; (6) removal of sub-mucous fibroid tumours per vaginam; (7) removal of uterine polypi; (8) amputation of neck of the womb; (9) hysteropexy, or ventro-fixation of the womb; (10) operation for ruptured perinæum and for prolapse of uterus and vagina; (11) curetting for endometritis and incomplete abortion; (12) exploratory operations (abdominal sections); (13) other operations. (1) Thirty-one patients were operated upon for the removal of ovarian tumours, of these six died, giving a mortality of 19·3 per cent. (2) For the removal of diseased appendages, twelve patients were operated upon, of whom one died, giving a mortality of 8·3 per cent. (3) It is stated in a note on page 14 of the tables of Drs Fenton and Eden, that six patients were operated upon for extra-uterine gestation; but on examination of the tables we find only five described as such, and one of these with insufficient reason; of the four remaining, in two, this condition was free from danger and would, probably, have been recovered from without operation; all these cases recovered. (4) Abdominal hysterectomy for fibroid tumour of the womb was undertaken in seven patients; of these six died, giving a mortality of 85·7 per cent. (5) The abdomen was opened for the removal of fibroid tumours in two patients; both recovered. (6) In two cases sub-mucous fibroids were partially removed by the vagina; both died of septicæmia—one of them being septic on admission. (7) In twenty-five patients fibroids and other polypi were removed; one died. (8) There were five cases of amputation of the hypertrophied cervix—one being cancerous; all recovered. (9) In nine cases hysteropexy, or ventro-fixation, was undertaken for displacement or falling of the womb; two of these died, giving a mortality of 22 per cent. (10) Sixty-one patients were operated upon for rupture of the perinæum, or prolapse of the uterus and vagina; by repairing the perinæum and narrowing the vagina, all recovered. (11) As regards curetting of the uterus, we have not obtained the number of patients in whom the operation was performed, but we find that death followed this operation in three cases. (12) Exploratory incision was performed in nine cases. In five of them no disease was found, or no organ so diseased as to require removal; four died, giving a mortality of 44·4 per cent. (13) We have no information about the other minor operations, but one death from pneumonia followed an operation for piles.

Of the twenty-six deaths after operations in the hospital, we are opinion that twenty were due to septicæmia, or were accompanied by symptoms of septic poisoning. Amongst these we include the cases which died with peritonitis.

On looking over these facts, we cannot help observing that the mortality from ovariotomy, hysterectomy, hysteropexy, and exploratory incisions is high. That from ovariotomy, 19·3 per cent., is nearly double the average mortality after this operation (question 1056). That after hysterectomy, 85·7 per cent., would, if it were general, be prohibitive of this operation, especially when it is borne in mind that fibroid tumour is rarely fatal.

Hysteropexy is an operation done for displacement and falling of the womb—conditions which give rise to much discomfort and suffering, but which do not threaten life; the mortality of 22 per cent. is high, and leads to the conclusion that this operation should only be done rarely and in very exceptional cases.

With regard to the exploratory operations, we have already observed that in five of the eight cases no disease was found, or no organ sufficiently diseased to demand removal. Circumstances such as these with a mortality of 44·4 per cent. render recourse to this method of diagnosis unjustifiable.

Curetting the cavity of the uterus, when done with care, is an operation practically free from danger. The occurrence of three deaths after it forces us, therefore, to the conclusion that due care was not taken to insure the conditions necessary for success.

As regards the results of the removal of the uterine appendages, myomectomy, removal of sub-mucous fibroids, removal of uterine polypi, amputation of the neck of the womb, operations for ruptured perinæum and for prolapse of the uterus and vagina, they are similar to those obtained in other institutions; all these operations, with the exception of oophorectomy and myomectomy, being accompanied by a minimum of risk.

It should be observed that the figures given above differ materially from those furnished by Drs. Fenton and Eden, and also from those contained in Dr. Parkes' letter of February 6th. As regards the divergences from the figures in the tables, drawn up by Drs. Fenton and Eden, it may be said that the amount of time that the staff had in which to furnish them was very limited, and that, therefore, perfect accuracy could scarcely be expected. There are, however, certain divergences due to differences of principle in stating results. With the principles enunciated by some other members of the staff in their evidence we cannot agree.

As to the divergences from Dr. Parkes' letter it is to be noted that he simply copied from the certificates of death furnished to the registrar of deaths by the resident medical officer of that hospital, and that except in regard to the cases of scarlet fever above referred to, none of his figures are over-stated to the detriment of the hospital, but rather understated to its advantage.

In referring to the deaths which occurred in the hospital, we have stated our opinion that a large number (20 out of 26) was due to septicæmia, and we must add that septic temperatures were not infrequent amongst the cases operated upon which ultimately recovered. This applies to cases of minor operations as well as to those of more serious character. As it has a bearing upon the general sanitary state of the hospital, it should be noted that septic temperatures did not prevail in the practice of every member of the staff.

The medical members of our committee inspected the wards of the hospital under the guidance of Dr. Fenton, and as it seemed that in some of the wards the cubic space allotted to each bed was insufficient, we requested Dr. Fenton to supply us with the exact data on this head. These he obtained for us, and we find that the cubic space per bed varies from 776 to 1,270 cubic feet. Seven wards containing thirty-three beds allow less than a thousand feet per bed. This is an amount which is considered insufficient even for persons in health. The largest space which is alloted to any bed in the hospital, viz:—1,270 cubic feet (with the exception of the isolation ward) is barely sufficient to satisfy the hygienic requirements of sick persons.

In regard to the administration of the hospital, we consider there is a great deal to be desired. It is obvious that the ultimate responsibility for the management should rest on the committee, who appeal to the public for funds to support their institution, that is the Committee of Management.

The bye-laws of the hospital provide for its management by two committees: (1). The Committee of Management, which held its meetings monthly, seems to have been well attended. It is obvious, however, that a meeting once a month is insufficient to exercise any adequate check on the daily routine of administration. (2). The House Committee, which met weekly, was practically the executive of the hospital. It is to be observed that on some occasions the treasurer was the only member who attended, while on other occasions only two or three were present. These committees were formed of non-professional and professional men: whether such a mixed committee is the best constitution for the management of a hospital is a matter of opinion, but we consider that such scanty attendance as noticed in regard to the House Committee renders management by committee ineffective.

We are further of opinion that owing to want of system there has not been proper control of the medical staff by the managers of the hospital. No doubt the law provides for procedure as to death certificates, but to give confidence to the public we suggest that in addition to the death certificates required by law there should be a post-mortem examination made by an independent authority who should be a pathologist elected by the Committee of Management. A post-mortem examination should be made in every case unless objection is offered by the friends of deceased. As a general principle of hospital management the pathologist should be responsible only to the committee of the hospital; notes of each case should be entered in a book, no erasure or alteration being made by any of the staff without the express sanction of the committee.

Want of organisation is to be noticed in regard to the absence of direct reporting as to the state of the hospital. The late resident medical officer stated that while he was supposed to be responsible, he did not report, but would mention defects such as drainage to the treasurer and medical staff "in conversation." This is obviously a serious matter; there should be some competent person intimate with every detail of the hospital and in a position to report and advise upon internal management, such as the condition of drains and appliances of all descriptions, and he should be in every respect the confidential adviser of the Committee of Management.

The medical records of the cases have not been kept in a completely satisfactory manner, and in our opinion there should be an independent registrar to keep a clinical record of every case admitted into the hospital.

We desire to place on record that we received every assistance in our inquiry from Mr. Wright and the secretary on behalf of the committee and, with one exception, from the medical staff of the hospital.

